# A Case for Openness – Book Publishing and the Role of Amazon

**DOI:** 10.1007/s40319-022-01159-w

**Published:** 2022-03-02

**Authors:** Antje Kreutzmann-Gallasch, Simone Schroff

**Affiliations:** 1 Ph.D.; Lecturer, Deakin Law School, Melbourne Burwood Campus, Melbourne, Australia; 2 Ph.D.; School of Law, Criminology and Government, University of Plymouth, Plymouth, UK

**Keywords:** Article 102 TFEU, EU competition law, Interoperability, Abuse of dominance, Amazon, e-book

## Abstract

The presence of Amazon is ubiquitous, especially in the online bookstore and e-book market. The introduction of the “Kindle” further cemented Amazon’s dominant position and business model in the market, having negative implications for authors, publishers and consumers. Publishers have less control over setting the price to have access to Amazon’s customer base. This will affect the authors’ remuneration and attribution. On the other hand, Kindle users are locked-in consumers, limited to Amazon’s e-book offering. This not only affects consumer choice but also reinforces Amazon’s market power due to the significant network effects. The European Commission attempted to increase competition in the e-book market by banning most-favourite-nation clauses, but this has seemingly failed. This article advocates for enforcing the Kindle’s interoperability with the e-book formats of other e-book providers. The proposed approach is beneficial for publishers as well as consumers. It safeguards copyright aims while alleviating the contractual constraints imposed by Amazon. Furthermore, consumers would benefit from broader flexibility when using their Kindle, allowing them to store and read e-books from the provider of their choice.

## Introduction

In 2017, the European Commission and Amazon agreed on conditions imposed on the largest trade book publishers to ensure competition in the e-book market.^1^ It was the culmination of a long-running dispute between Amazon and the largest book publishers about the latter’s loss of pricing control. One of the main reasons for opening the formal investigations in 2015 was that Amazon used most-favoured-nation (MFN) clauses also known as parity clauses in its e-book distribution agreements with e-book suppliers.^2^ Amazon used various types of MFNs to control, for example, the availability, specific features, format and prices of e-books.^3^ While MFNs can foster innovation as they allowed Amazon to recover its cost, they also had an anticompetitive effect by harming e-book distributors and creating a barrier to entry for competing e-book providers.^4^ After examining the anticompetitive effects of the MFNs, the Commission considered that a five-year ban on including MFNs in new agreements or enforcing such clauses in existing agreements would be sufficient to boost competition in the e-book market.^5^ The key condition, the ban of most-favoured-nation clauses is expiring in 2022, but the underlying issues have not been resolved. Publishers are still facing the same threat: the overwhelming market power of Amazon. The underlying dynamic is driven by copyright law, the very same law that used to underpin publisher control in the first place. The solutions adopted under copyright to limit online piracy, especially the strong protection and widespread use of highly restrictive digital rights management software, create major obstacles for consumers to switch between providers while limiting consumer behaviour beyond what copyright envisions.^6^ This article proposes a solution to the problem through competition law, prioritising consumer choice to enable consumers to read new innovative e-books, which are not yet available, on their electronic reading devices in the future.

This article will first outline how digital technology, focusing on e-books, has changed the distribution arrangements within the publishing industry. It will pay particular attention to the effect this had on publishers and the consumer. It will show that the dominant role of Amazon has changed the industry dynamics and needs to be addressed by critically evaluating the role played by competition rules and the use of digital rights management (DRM) software. It then proposes a solution by targeting Amazon’s dominant position in the marketplace and the pivotal role the Kindle environment has in this. The authors conclude that there is one effective route to resolve the current imbalances to the benefit of right holders and consumers while strengthening competition. That solution is to open up the Kindle by enforcing interoperability through Art. 102 Treaty on the Functioning of the European Union (TFEU).

## The Undermining of Control: When Copyright Meets Strong Market Actors

Copyright law is the foundation of the creative industries. It allows for creative labour to be turned into a commercial product, turning the intangible into a valuable asset that can be sold.^7^ It gives the creator something to sell,^8^ which is a prerequisite for the creative industries to operate efficiently. More formally, within the EU, one core aim is economic: provide the creator and those intermediaries working with them the exclusivity required to recoup their investments.^9^ For this to work in practice, copyright law is premised on contractual freedom: the right holder is free to exploit their works as they see fit, entering into agreements with others to maximise the value of the work. The only inherent restrictions are copyright exemptions that in practice are limited to non-commercial, public benefit uses rather than allowing larger scale commercial interventions (see the Berne three-step test)^10^ and certain contractual provisions between authors and commercial intermediaries.^11^

One key assumption in contractual freedom is that the right holder expects at least some degree of price control. Unfair market power affects this basic logic as individual authors are often faced with a bottleneck of strong commercial intermediaries who are essential to make their work a commercial success, leaving them unable in practice to extract fair deals.^12^ Copyright has reacted through contractual safeguards enshrined in EU and national law, including guaranteed and unwaivable remuneration rights.^13^ However, the internet age has brought the entry of entirely new stakeholders, epitomised by Amazon, which now dominates book sales, especially e-books.

Like other creative industries, book publishing has been significantly affected by the digital revolution in producing and distributing books. While a full discussion of the changes is beyond the scope of this article,^14^ the way the final product, the book, is accessed by the consumer has been transformed. Traditionally, the publisher could only reach the consumer through brick and mortar booksellers, either by supplying them directly or through a wholesaler acting as a warehouse intermediary.^15^ To support sales, the publisher cooperated with the booksellers to ensure its books were in stock and advertised – making significant concessions to booksellers by absorbing the risk involved.^16^ The internet has changed this relationship as traditional booksellers came under pressure from online retailers.

Online retailers are able to outcompete brick and mortar booksellers due to the choice they offer. The attractiveness of a bookstore is defined by its the location, ambience and especially the books they stock.^17^ The internet has not changed this, although the meaning of the individual criteria has evolved. Location has in the past been a proxy for convenience or easy accessibility, traditionally a payoff between a more expensive central location and the higher rents this entails. In the online world, this is replaced by having an affordable and timely delivery service and, therefore an effective logistics network. Ambience in a bookstore refers to the style of the store, but online it refers to homepage design and ease of navigation. The real differentiating criterion, however, is the third key attractiveness factor: choice. To be attractive to the consumer, a store needs to offer the book the consumer wants with minimal delay. This means in practice the larger the stock, the more attractive the offering. Traditional booksellers are limited in what they can stock by the size of their stores: the better the location, the more expensive the rent and therefore the smaller the stock held in store. Further, the commercial life or “shelf-life” of printed books is limited, with less than one quarter of published books being available for more than 12 months and 90% of tangible books are only available in the first two years of their publication. Furthermore, most of these books will only be re-published after the expiration of their copyright protection.^18^ In contrast, e-books cannot go out of print; they are digitally generated and stored. Online stores operate warehouses, making the storage cost per item significantly cheaper. The strong appeal of online shopping is epitomised by the first mover in the field: Amazon. It offers easy and timely access to books and stocks a very wide range from different publishers and as a result is often more attractive to the consumer than brick-and-mortar booksellers.

Amazon effectively used its strong position as an online bookseller to corner the new e-book market, capitalising on the difficulties other market actors faced.^19^ Amazon benefitted from traditional booksellers not moving online on a large scale – allowing it to develop its brand as a one-stop-shop for all kinds of books. Publishers also face significant barriers in building their own attractive online presence. In particular, their offer is limited to their own books and therefore inherently limited in choice. This means that the consumer would have to go to several online stores to meet her preferences, increasing the transaction costs significantly. Most importantly though, Amazon took the lead in building the e-book market by introducing the most popular and affordably priced e-book reader (the Kindle)^20^ while licensing content as e-books on a large scale from a variety of publishers. In theory, this should have benefitted publishers as it opened an additional distribution channel. However, the particular approach Amazon (and other providers later on) chose prevented the development of a competitive market.

The market in e-books shows limited competition between providers due to consumer lock-in. All major providers such as Amazon use proprietary formats and strong digital rights management-based closed environments. The combined effect is that moving content across providers is often impossible or at least very difficult and more importantly, illegal under copyright law.^21^ As a result, the consumer often chooses one provider, most likely the one with the widest selection of books. In other words, the publishers and other competitors are not able to provide a universally attractive online distribution option to the consumer. Instead, Amazon has become the one stop shop for consumers with extensive market power.

### Effect on the Publisher: Price Control

Amazon identified the potential of e-books early on as a business opportunity, but its approach undermined the publishers’ business models more widely. Books are traditionally sold based on a windowing strategy: expensive hardcover books are released first, followed later by cheaper softcover versions. Books are sold to the bookseller using the wholesale model: the publisher sets a recommended retail price (RRP) and then sells the book to the distributor with a discount, often between 30 and 50%.^22^ Booksellers are free to sell at a lower price,^23^ but their scope to do so is limited in practice as costs are significant, including running the store and paying employees among others.^24^ As a result, the discount is essentially the booksellers’ margin – they have no interest in going below the RRP,^25^ which means that the publishers indirectly control book prices.

The conventional business model was undermined because Amazon’s position in the market is fundamentally different. Amazon offers a wide variety of products and its reputation as a bookseller supports attracting customers more broadly to shop on its platform. E-book availability is therefore key not only because it adds incremental sales in addition to substituting for analogue book sales,^26^ but because increases traffic to the platform more broadly. It targeted the pricing of books, discounting not only analogue books at higher, more consistent rates but pushing e-book prices down by even selling them below cost.^27^ This strategy affected especially popular books and new releases, the most important ones for the publishing industry due their higher margin and volumes.^28^ While each sale is strictly speaking a loss for Amazon, the stronger overall market position made it viable.^29^

However, the effect this had on publishers was profound as publishers lost de facto price control and with it control over their business model. While the lower prices benefit consumers, it exacerbates the pressure on other booksellers who are not able to work within these squeezed margins.^30^ As Kirkwood summarises succinctly, the publishers feared that the lower price was affecting their hardcover sales, changed consumer expectation of what an e-book should cost, accelerated the decline of brick-and mortar booksellers (and with it a key showroom for their books as consumers can see them without explicitly searching for them), and the threat of disintermediation as Amazon was moving into the publishing business itself.^31^ The obvious solution to push e-book releases back to be in line with softcover ones has not worked as it fuelled online piracy, customers were worse off and overall sales declined.^32^ Tensions were further amplified when the Amazon library offered e-books without the explicit permission of right holders. Amazon saw it as permissible since publishers were paid as usual, but publishers rejected the loss of control.^33^ The result was a stand-off between the publishers and Amazon on the chosen battleground of e-book pricing.

### The Empire Strikes Back – And Does Not Win

The publishers identified control over end prices as the key remedy to their loss of control and tried to re-negotiate their contracts with Amazon to switch to an Agency model.^34^ In this approach, the publisher sets the retail price and the bookseller gets a percentage fee – usually around 30%. Amazon refused, triggering disputes with major publishers which spilled into the open. Most famously, the publisher Hachette had their pre-order option removed, deliveries delayed and rebates revoked – seriously affecting its sales.^35^ Events also made it clear that the publishers were subject to a collective action problem as those publishers not challenging Amazon would benefit from the fallout.^36^

The collective action was resolved once a second major corporation entered the e-book market: Apple. The major publishers colluded with each other in their negotiations with Apple when it introduced its iBookStore for the iPad. Rather than using the wholesale model, they switched to the agency model in combination with a most-favoured-nations clause which gave Apple the security that any e-book offered in their store was not more expensive than on Amazon.^37^ This in turn significantly reduced publisher income – a move which can only be understood as an investment to force Amazon to follow suit.^38^ Most importantly though, the collusion was found to be anticompetitive and resulted in the prohibition of both the agency contracts and retaliation for several years as well as a fine for Apple due to the nearly instant higher prices for consumers.^39^ In 2017, the EU took further action by also banning Amazon to use MFNs until 2022 to provide space for other platforms to enter the market.^40^

As far as the Commission was concerned, the dispute was brought to a hold. Amazon backed down in its dispute with Hachette after it became clear that all six major publishers were going to push for the change.^41^ Indeed, Amazon seems more willing to accept the agency model but without raising its prices.^42^ Relations remain uneasy, Amazon is still exceedingly powerful and once the prohibition of MFNs expires in 2022, it is likely that the dispute will flare up again. Equally important though: the current stalemate does not address the interest of the consumer. Consumers are still locked into the e-book provider’s system. In addition, when consumers purchase e-books, they no longer buy a tangible product: instead, they access e-book files in a software environment, most commonly through an app or a dedicated reader. The consumers’ experience and behaviour are filtered through the software environment and determined by either the right holder or manufacturer. As a result, they can limit the user’s actions. The software environment itself is DRM protected^43^ and as a result, breaching the restrictions amounts to copyright infringement.^44^ At the same time, exemptions available for DRM removal or modification do not cover most of the usual copyright exemptions.^45^ Reading the same book analogue is outside of copyright law while the digital version is subject to a more stringent regime than envisaged by the law – a state of affairs which cannot be justified under copyright law.^46^ The expectation for e-commerce in relation to online content was that providers would compete with each other, offering a variety of prices and privileges to meet consumer demand closely, but this has proven impossible in practice.^47^ As a result, a more comprehensive solution actively fostering competition between e-book providing platforms is required to give consumers choice not only where to buy their books but also under what conditions.

## Competition Law Round II: Targeting Openness, Not the Contracts

So far, the Commission’s preferred approach has been to focus on contracts and remove any contractual clauses which have a foreclosing effect for new market entrants.^48^ However, this section will show that a more fruitful approach is to address Amazon directly through Art. 102 TFEU, focusing on harmful effects of its dominant position. While the solution proposed here focuses on Amazon, it has wider applicability as implementation across the sector would benefit both competition and consumers in the long-term by removing the distortion introduced by the DRM-based lock-in of consumers.

### Introduction and the Relevance of Art. 102 TFEU

Amazon’s market power and distorting effect is based on its gatekeeper function. Traditionally, the publisher had the gatekeeper role between the author and the bookstore. Now, publishers must share this role with e-book providers who control the operating system through which authors and publishers must go to reach their audience – the reader. This puts an e-book provider in an advantageous position: it allows them to control market access and therefore puts pressure on traditional publishers by dictating their terms and conditions and negotiate lower wholesale prices. Traditional publishers do not have a choice if they wish to offer their product to a wider audience and increase their sales.^49^ Not offering an e-book version of a book would result in revenue losses which may harm the author and publisher who will miss out on royalties and incremental sales. One must bear in mind that the e-book sector is no longer a niche sector; the global revenue forecast for e-books for 2021 is more than 15 billion US$ and expected to grow to almost 18 billion US$ by 2025.^50^ Amazon’s involvement in the “publishing wars” shows its prominent position in the e-publishing market. There are two options available to address an imbalance between the involved parties: economic regulation or competition law.

Economic regulation is considered necessary where competition alone is insufficient to address market power.^51^ Regulatory bodies used economic regulation, for instance, in markets with one provider (monopoly) to introduce competition by removing barriers to entry and increasing access for new entrants.^52^ Utilities sectors, such as electricity, gas, post, and telecommunications, have been prominent examples where economic regulation was used to liberalize or privatize the market while ensuring consumer protection and the continuous provision of essential services through the imposition of universal service obligations on the incumbent.^53^ However, the e-book sector is not a monopolist market. In addition to Amazon, e-books are also offered by, for example, Barnes & Noble, Google, Apple, Rakuten and Hachette. While not all of them operate in the same geographic market, they all compete with Amazon in Europe, the United States and China.^54^ In other words, Amazon does face some competition.

*Ex ante* regulation concerning market power can also be used in a competitive market; however, this might lead to a distortion of competition rather than an increase in competition between different providers.^55^ Introducing regulation in a competitive market removes the incentive to offer lower retail prices. Littlechild points out how regulatory intervention in the competitive UK retail energy market has decreased consumer protection as lower tariffs at retail level were abolished, while the profit margins of the six largest electricity providers increased by 10% at the same time.^56^ In a competitive market, economic regulation should therefore be considered very cautiously when tackling market power. Competition law is generally considered to be the more effective tool and better suited to address competitive constraints. This applies not just to traditional markets but also to digital markets and the e-book sector as being part of online platforms.^57^

Based on this, it is argued here that Amazon’s position can be challenged through competition law and in particular Art. 102 TFEU.^58^ It prohibits a dominant undertaking abusing its market power to impose unfair conditions on another party.^59^ In this context, it has to be proven that Amazon uses its position in the e-book sector to prevent effective competition. Nonetheless, not every dominant position is unlawful and, therefore, prohibited. The dominant undertaking must be in a position of economic strength that hinders or distorts effective competition and is incompatible with the internal market.^60^

### Relevant Market, Dominance and Abuse

Amazon’s position in respect of publishers, authors and consumers must be discussed in light of the relevant market.^61^ Defining the relevant product market is a crucial step: a too narrowly defined market gives the impression that an undertaking is dominant when it is not, whereas Art. 102 TFEU would not be applicable if the market is defined too broadly. Amazon sells a wide range of products through its online platform, offering entertainment services and its own electronic devices. Books, both print and e-books, are not among the top 10 most popular products, but it is a product category that offers a high profit margin and is therefore attractive.^62^ More importantly, the relevant product market as envisioned by Art. 102 TFEU consists of interchangeable products that are comparable by price and characteristics and intended for a similar use.^63^

The relevant product market is restricted to the e-book sector. E-books are not a substitute for print books. They must be distinguished from print books because of their distinctive features. Probably the most important difference is that e-books can only be accessed and read through an e-book reader or other electronic devices.^64^ The relevant product market is a two-sided market. It encompasses both sides, the authors/distributors as well as the end-consumer who purchases the e-book from the provider. Authors and publishers depend on the service of e-book providers to convert their work into an appropriate format or at least on their distribution services to reach the end-consumer. For the consumer, the more books are offered through the respective platform, the more appealing the platform will be and vice versa.^65^ E-book providers act as intermediaries between the author and publisher on one side and the consumer on the other side. Any solution adopted therefore has to take into account both sides of the market.

The determination of the geographic market is another crucial component in establishing the relevant market. It is accepted that the geographic market comprises an area in which the undertaking offers the product and “where the conditions of competition are sufficiently homogenous.”^66^ Amazon operates digital stores in seven Member States and the United Kingdom.^67^ Access to Amazon’s stores and the ability to download e-books is not restricted to consumers of that respective country. Therefore, the geographic market should not be interpreted too narrowly but rather include the EEA market and the United Kingdom.^68^

Amazon also holds a dominant position in the relevant markets. Dominance implies that the company has a special position in the market but having a gatekeeper role alone does not suffice to establish dominance. In *United Brands v. Commission*, the Court of Justice, developed the relevant test and clarified that dominance requires a position of economic strength that allows the concerned undertaking to interfere with the market and hinder effective competition. The Court further clarified that dominance generally “derives from a combination of several factors which, taken separately, are not necessarily determinative.”^69^ Market shares are generally regarded as an “easily available proxy for the measurement of market power.”^70^ Even though market shares alone are not sufficient to determine a dominant position, the European Court of Justice and the General Court have held that they are a “significant” indicator whether or not an undertaking holds a dominant position.^71^ In *Akzo v. Commission*, the ECJ clarified that having a market share of at least 50% can be evidence for a dominant position,^72^ while in other cases it was presumed that a dominant position exists where the undertaking concerned has a market share between 70 and 80%.^73^ More than a decade ago, the European Commission set out that a market share of 50% or higher may serve as an indicator for a dominant position.^74^ In *Hoffmann-La Roche v. Commission* the ECJ further elaborated that a high discrepancy between the market share of the undertaking and the competitors may be another indicator for the lack of effective competition.^75^

In Germany, France, Spain and Italy, the four largest Member States in terms of population, Amazon had a market share in the e-book sector between 62 and 73% in 2019 which is a strong indicator for Amazon’s dominant position. In the United Kingdom the market share was with 84% even higher.^76^ Even though the UK is no longer bound by Art. 102 TFEU, the working of Sec. 18(1) and (2) of the UK Competition Act 1998 are practically identical to Art. 102 TEFU, with the exception that the scope of the Competition Act is restricted to the United Kingdom. In comparison to Amazon, the second strongest providers in each of the above-mentioned countries recorded notable fewer purchases in the same period. The user share of Amazon’s closest competitors ranged from 32% in Spain (Case del Libro) to 18% in the United Kingdom (Google Play Store).^77^ This puts Amazon in a position of economic strength and makes it difficult for publishers and authors to avoid Amazon as distribution platform. Thus, it can be concluded that Amazon holds a dominant position in the e-book sector.

Additionally, the dominant position can be manifested by economic or legal barriers that prevent new firms from entering the market or existing competitors from expanding.^78^ Amazon’s business model is based on vertical integration; Amazon acts simultaneously as publisher and retailer^79^ and attracts a large customer base as a brand, which makes Amazon an essential business partner for content creators. Creating such a network is expensive and may result in higher unit costs.^80^ However, Amazon benefits from economies of scale in the e-book market. Once an e-book has been produced, the average costs of producing an additional unit will fall as output increases.^81^

Amazon’s dominant position is further consolidated by the lack of interoperability of their e-books.^82^ Amazon (like other providers) relies on DRM protection and proprietary formats to ensure that its e-books cannot be transferred to a competitor’s device, for example, to Apple’s iBookstore. Instead, the costumer must either purchase an e-book reader (Kindle) or tablet (Fire) from Amazon or download the free Kindle app onto their smartphone, tablet or computer.^83^ There is third-party software available which facilitates transfers, but its use is infringing under copyright law since it removes the DRM and copying restrictions. As a result, the consumer is locked into an environment which enables Amazon to ascertain control – Kindle users have no choice but to purchase e-books from Amazon; the lack of interoperability is the major factor for Amazon’s dominant position in the e-book sector and the existing competitive constraints within the market.

Gilbert argues that consumers would “abandon the Kindle platform” and switch to a different e-book provider if Amazon would not offer competitive prices.^84^ However, switching comes with costs. Consumers would have to purchase a new e-book reader from a different provider and would lose access to e-books they have already purchased. It would also require that they invest time in searching and familiarizing themselves with a new system. In addition, the ability to share Amazon libraries with another user creates an additional disincentive to switching. Empirical research assessing the switching behaviour of consumers across several sectors has shown that unless the financial gains are clearly communicated, elderly consumers and consumers with high-income (the most profitable ones) are less likely to switch.^85^ It should also be noted that there is no difference between the relevant product market consisting only of e-books purchased from Amazon or a product market that also includes e-books from alternative platforms. Even under the wider approach, competitive constraints would not be removed as Amazon still holds a dominant position in the e-book sector.^86^ The Commission took the view that the lack of interoperability “does reinforce Amazon’s market power vis-à-vis its competitors since consumers willing to move to another platform are likely to face switching costs and may therefore effectively remain locked into Amazon’s closed ecosystem.”^87^ These network effects make it easier for Amazon to maintain or expand its dominant position but more challenging for new or alternative e-book providers to enter the market or to grow respectively.^88^

Article 102 TFEU does not prohibit dominance per se as only the abuse of the dominant position is prohibited. A dominant undertaking is entitled to engage in conduct that is lawful and compete with other firms on its merits. Lawful conduct must be distinguished from abusive behaviour, that is, behaviour preventing competition.^89^

Amazon took advantage of its gatekeeper position and engaged in discriminatory abuse. Amazon used various price and non-price parity clauses and other most-favoured-nation clauses in its distribution agreements to prevent book publishers or intermediaries to offer the same product to a different e-book provider at a lower price or to offer it to a competitor first.^90^ In 2015, the European Commission initiated proceedings against Amazon and later expressed its concerns regarding the compatibility of such MFN clauses with Art. 102 TFEU in its preliminary assessment pursuant to Art. 9(1) of Regulation (EC) 1/2003.^91^ The Commission found that the use of MFN clauses affects innovation as they disincentivise smaller firms or new entrants from developing new business models to distribute e-books or develop new and enhanced features. Under their agreement with Amazon, they are contractually obliged to notify Amazon of their idea, develop identical features that work with the Kindle format. As a result, Amazon would be able to free-ride on their ideas, while the smaller firms cannot recover their costs and may potentially even be forced to exit the market.^92^ The Commission found that Amazon’s choice of price parity clauses guaranteed that Amazon would not charge a higher retail price than any of its competitors.^93^ Consequently, the incentive for consumers to switch to a new e-book retailer is lower.^94^ Overall, the Commission concluded in its Preliminary Assessment that each of these clauses constitute an abuse.^95^

MFNs adopted by digital platforms are a more recent phenomenon, but they are here to stay. Nonetheless, they have been under the scrutiny of the Commission and national competition authority for some years now. It appears that the authorities favour Art. 101 TFEU in their MFN investigations.^96^ The Amazon e-book inquiry under Art. 102 TFEU was the exception rather than the norm.^97^ Although Art. 101 TFEU appears to have been the preferred approach by the authorities, Art. 102 TFEU is not a less effective tool to investigate MFNs in digital markets as the Amazon e-book case has shown. More recently, the Commission relies more on Art. 102 TFEU. For example, in the *Google Shopping* case the Commission found that Google abused its dominant position since its search engine favoured its own comparison shopping service over services offered by competitors.^98^ Akman argues that the assessment of MFN in digital markets “under Article 102 may be legally more appropriate and sound.”^99^ So far, the Commission has not committed itself to one method but follows a case-by-case approach.^100^ At present, the Commission is investigating Apple’s gatekeeper role in relation to its mandatory in-app distribution system under both Arts. 101 and 102 TFEU.^101^

The Commission adopted in its 2017 *Amazon e-book* Decision that parity clauses in contracts can no longer be enforced and ordered Amazon not to include such clauses in any new e-book agreements for the next five years.^102^ The Commission argued that by removing parity clauses, publishers would have a greater incentive to produce e-books in multiple formats and offer them to other e-book providers, and competitive e-book providers are less motivated to use or develop their own e-book format.^103^ It appears that the adopted measures have not had a substantial impact. Amazon has managed to further consolidate its position in the European e-book sector.^104^

In the light of the above discussion, it becomes clear that there is a need for additional action as currently consumers do not benefit from effective competition and authors and their publishers have no choice but must offer their products through Amazon to sell their work to a wide audience.

Based on the well-established principle that a dominant undertaking “has a special responsibility not to allow its behaviour to impair genuine, undistorted competition on the internal market,”^105^ a more effective approach to address Amazon’s anticompetitive conduct in the e-book sector would be to increase the interoperability between the various formats by ordering Amazon to open the Kindle to other e-book formats and to ensure that Amazon’s e-books can be read on alternative devices. Since in practice, the feature that interferes with competition is the use of restrictive e-book formats. By restricting interoperability, Amazon abuses its intermediary position and therefore infringes Art. 102 TFEU.

### A Novel Approach: Enforcing Interoperability

Enforcing interoperability, starting with Amazon, would give consumers the choice to purchase their e-books from different providers while still being able to use their Kindle. Authors and publishers would be given a greater choice with whom they want to enter into an agreement and their content would still be accessible to a wide readership through a platform of their choice. As shown in this paper, Amazon’s lock-in model prevents consumers from reading alternative e-book formats on the Kindle. Being able to read the same e-book on different devices, for example, through the Kindle App on the smartphone or tablet does not change that; the access is still exclusively restricted to digital books downloaded from Amazon’s platform. It does not give end-consumers the possibility to choose freely and switch from one e-book provider to another. Restricting the reader to one platform also limits their choice of books – not all books are available as e-books on Amazon. Nihoul has reviewed Commission Decisions concerning Art. 102 TFEU and showed that limitation of choice is a preferred tool adopted by dominant undertakings.^106^ Consumer welfare is not just concerned with low prices, but in the context of Art. 102 TFEU also comprises consumer choice.^107^

In the e-book investigation, it has been suggested that the Commission should consider imposing additional obligations on Amazon due to the closed ecosystem of its Kindle reader and e-book formats.^108^ The lack of interoperability of the e-book reader and e-books concerns Amazon’s technical know-how and intellectual property rights. Understandably, a firm has an economic interest to protect its technological knowledge. Intellectual property rights give an owner the right to exclude others to collect or exploit the fruits of their work.^109^ Nonetheless, intellectual property rights are not absolute. The refusal to grant access to the Kindle format may amount to an abuse of dominance in “exceptional circumstances”.^110^

The lack of interoperability is not a new phenomenon. One of the most prominent cases is the *Microsoft* case.^111^ Microsoft had refused to disclose technical information that would have allowed an external firm to develop an alternative work group operating server system,^112^ which could have been seamlessly integrated into the Microsoft system.^113^ The Commission found that the refusal to supply the proprietary information was a breach of Art. 102 TFEU (then Art. 82 EC).^114^ On appeal, the Court of First Instance (CFI, now General Court) upheld the Commission Decision.^115^ The CFI used this opportunity to refine the test that the Court of Justice had laid out in *Magill.*^116^ According to the CFI in *Microsoft*, the following circumstances must be met for “exceptional circumstances” to exist:

- The refusal relates to a product or service indispensable to the exercise of a particular activity on a neighbouring market;
- The refusal is of such a kind as to exclude any effective competition on that neighbouring market;
- The refusal prevents the appearance of a new product for which there is potential consumer demand;
- The refusal is [not] objectively justified.^117^

It will now be shown that the lock-in effect created by Amazon satisfies the conditions and, therefore, constitutes an abuse under Art. 102 TFEU.^118^ Regarding e-books, interoperability would require interoperability between hardware and software. So that consumers who own a Kindle e-book reader could purchase e-books from competing providers. It would also allow consumer who own an alternative e-book reader to download e-books from Amazon.^119^ It must also be indispensable^120^ for competing firms to offer their products in the secondary e-book market. In *Microsoft*, the CFI declared that interoperability was indispensable because of Windows “quasi-monopoly” position on the PC operating market which made it impossible for competitors to promote their products if they are not compatible with Windows.^121^ In *Microsoft*, the CFI interpreted “impossibility” as without gaining access to the proprietary information, it would not be “commercially viable” for the other company to compete with the dominant undertaking.^122^ It could also be argued that Amazon’s position in the e-book market is less dominant than Microsoft’s since there is some level of competition in the e-book market.^123^ Yet, Amazon’s market power has been strengthened further by the lack of interoperability.^124^ Besides, Amazon is a highly vertically integrated undertaking with a strong presence in the e-book sector which has become an “unavoidable trading partner” in the e-book market.^125^ Due to its large consumer base, it is inevitable for publishers and intermediaries to enter into agreements with Amazon. Amazon can, therefore, offer a large variety of books which in turn attracts consumers.^126^ New entrants cannot viably provide their products, or it would be highly challenging for them to do so.

Next, the lack of interoperability must exclude any effective competition on the secondary market. Exclusion refers to the “elimination of effective competition” by “marginalizing competitors and preventing them from exercising any effective competitive pressure on the dominant undertaking.”^127^ Currently, it appears that while there are some other big players operating in the e-book sector, it does not put enough pressure on Amazon to have an influence on Amazon.^128^ Even with big players such as Apple and Google entering the market, Amazon was able to maintain its dominant position.

The third criterion of the test requires that the lack of supply prevents the emergence of new products, “which consist in limiting production, markets or technical developments to the detriments of consumers”.^129^ There is a low incentive to invest in innovation. Most e-books are formatted by publishers or intermediaries to ensure that the e-books are formatted at a high standard and protect the author’s control over the end-product.^130^ Developing new and innovative e-books with unique features that make them more interactive or have better illustrations is an expensive and lengthy process for publishers since they must ensure compatibility and the correct display of those new features across different devices.^131^ The lack of interoperability, therefore, not only prevents Kindle customers from accessing innovative e-books but also, and even more importantly, may prevent the overall creation of innovative e-books in the first place. Moreover, these adverse effects are reinforced by Amazon’s market power and the strong network effects. Accordingly, the lack of interoperability limits technical development to the prejudice of consumers.

And last, the refusal to licence must not be objectively justified. The General Court acknowledged that the refusal to grant a licence can be justified if there would otherwise be a negative impact on the dominant undertaking’s incentive to innovate. However, a negative impact on the undertaking’s incentive to innovate alone is not sufficient. The General Court seems to apply a balancing act between the procompetitive and anticompetitive effects.^132^ LaRouche points out that it would be difficult to decide this in favour of the undertaking concerned, as it would require to make future forecasts and that “innovation is unpredictable”.^133^

Hence, there appear to be “exceptional circumstances” that grant competitors the right to access proprietary information to obtain interoperability of software and hardware.^134^ Such a behavioural remedy would comply with the proportionality and necessity requirement.^135^ It would terminate the infringement, repetition would be unlikely and it would help to achieve effective competition.^136^ Additionally, this case would establish a legal precedent for other operators.^137^ Looking forward, addressing Amazon’s abuse of dominance in the e-book sector even has the potential to achieve standardization of the different e-book formats.^138^

The proposal also complies with the needs of copyright law to limit infringement through unauthorised copying and online sharing. Piracy is a key concern to the e-book industry because it is reflected in lost sales and therefore reduced income of right holders.^139^ This includes pirated copies being offered for free on torrent and piracy homepages, most commonly as PDFs but also in the open EPUB format.^140^ The problem is widespread and is especially prevalent among the young and the new generation of readers.^141^ Opening the Kindle is not actually removing the DRM, but instead ensures that other properly licensed providers can access the device. It therefore does not make piracy easier in any way. Furthermore, the particular way e-book distribution works in practice limits the impact of piracy. Amazon relies on direct licenses with right holders and its sharing features are limited. In addition, commercial success dictates working through popular platforms to reach the end consumer on a large scale, centralising distribution in the hands of a few platforms which are easier to police than the internet as a whole. Amazon’s large market share^142^ makes it the primary target for effective enforcement. Like other large platforms, Amazon has a well-established notice and takedown procedure and has proven responsive in taking down content.^143^ It also has an inherent incentive to cooperate with right holders, given its own Kindle store and its reliance on third-party content.^144^ Finally, locating an e-book on the homepage works via the Amazon proprietary search algorithm and therefore is under Amazon’s control.^145^ It can be used to prioritise legitimate content. In others, while the publishing sector is affected by piracy, the commercial impact is limited by structural factors that limit the commercial potential of infringing copies.

## Conclusion

The situation in the book publishing sector is characterised by Amazon controlling most of the e-book market. Amazon’s position is based on its popular, easy-to-use Kindle e-book environment, its wide selection of e-books and the lock-in of consumers who cannot move freely between providers. In practice, the market power this creates for Amazon has created a long-running conflict with the publishers and detrimental welfare outcomes for consumers alike. The publishers cannot forgo this distribution channel since the financial repercussion in terms of lost sales and royalties would be extensive. The controls exercised over consumer behaviour, build on the copyright-supported protection for DRM, undermining copyright as a whole by negating consumer rights through software restrictions going beyond what copyright envisioned. The balance of power between right holders and consumers and therefore the core of copyright law has been permanently shifted to the detriment of the consumer and society more widely. Competition law, in particular Art. 101 TFEU, has been used in the past but only created a stalemate, not an actual solution and it has not addressed the consumer’s lack of choice. It is therefore necessary to take a more comprehensive approach that aims at strengthening competition rather than simply maintaining current levels.

An effective solution is to address the role of Amazon under Art. 102 TFEU. Amazon is a dominant player in the market, it has abused its dominance, and a copyright compliant solution is available: the lock-in effect created by DRM. Competition law has the advantage that the creator will still be recognised and renumerated for their work.^146^ Yet, consumer choice can be enabled by gaining interoperability of the Kindle e-book reader and Amazon e-books. This would ensure that the Kindle reader, whatever the platform, is open to other formats, allowing consumers to buy their e-books wherever they wish without forgoing their existing collections and allowing non-Kindle owners to benefit from Amazon’s e-book catalogue. With consumers shopping around, competition between providers should also lead to terms and conditions more aligned with actual consumer preferences, such as staggered pricing for different levels of privileges. Furthermore, an open environment reduces the potential for abuse through Amazon’s bargaining power as publishers can offer viable alternatives. Finally, by opening up the dominant environment in the market, this can create a consumer expectation of openness, forcing other players to follow suit, reinforcing the cycle of competition, with benefits for all market actors.

## Footnotes

1 Amazon proposed commitments which were adopted by the Commission pursuant to Art. 9(1) of Regulation (EC) No 1/2003. European Commission, Commission decision of 4 May 2017 relating to a proceeding under Art. 102 of the Treaty on the Functioning of the European Union (TFEU) and Art. 54 of the EEA Agreement, Case AT.40153 – *E-Book MFNS and related matters (Amazon)*, C(2017) 2876 final, https://ec.europa.eu/competition/antitrust/cases/dec_docs/40153/40153_4392_3.pdf; Aaron McGrath, “Commitments – Commitments: Case Comp/AT.40.153 E-BOOK MFNs and related maters” (Final Commitments, 29 March 2017), published on 28.07.2017 https://ec.europa.eu/competition/antitrust/cases/dec_docs/40153/40153_4393_3.pdf.

2 Most-favoured-nation clauses (or so-called MFNs) cover a range of contractual terms used by one party to obtain an advantage (*e.g.* price benefits) from the other contractual party.

3 For an overview of all MFNs incorporated by Amazon into contracts with publishers and wholesalers, *see* European Commission, decision 2017 (*supra* note 1), paras. 22–38. Wholesale prices can only be set by parties where this process is not prohibited by domestic laws, European Commission, European Commission, decision 2017 (*supra* note 1), para. 21.

4 Johnson (2017), pp. 1151, 1173.

5 European Commission, decision 2017 (*supra* note 1), paras. 189–191, 193.

6 These debates are explained in detail below.

7 Searle and Brassell (2016), p. 10

8 Townley et al. 2019, p. 9.

9 Ramalho (2014).

10 For a detailed assessment, *see* Geiger et al. (2014).

11 These include moral rights but also remuneration rights which cannot be waived. It should be noted that most of these provisions are at the national rather than EU level.

12 Adeney (2006); Davies and Garnett (2016); Ricketson and Ginsburg (2006). Also, Directive (EU) 2019/790 of the European Parliament and of the Council of 17 April 2019 on copyright and related rights in the Digital Single Market and amending Directives 96/9/EC and 2001/29/EC, OJ L 130/92, chapter 3.

13 Adeney (2006); Davies and Garnett (2016); Ricketson and Ginsburg (2006).

14 For a comprehensive overview and analysis of the publishing industry, *see* Caves (2000); Thompson (2012); and Greco (2014).

15 For the detailed dynamics underlying the publisher-bookseller relationship, *see* Caves (2000), pp. 146–157; Thompson (2012), pp. 238–291; Barnett (2014); Garon (2013), p. 581.

16 Caves (2000), pp. 146–157; Thompson (2012), pp. 238–291.

17 Thompson (2012) 26–58.

18 Australian Government Productivity Commission, “Productivity Commission Inquiry Report” (September 2016), No. 78, pp. 130–131; *See also* Parc and Messerlin (2021), p. 613. It must be noted that tangible books may be out of print if they are either sold out or the publisher can – depending on contractual agreements between the publisher and author – declare a printed book out of print in circumstances where there is a lack of demand. For an overview of “out-of-print” clauses, *see* Columbia Law School, “Keep your Copyrights”.

19 Competition law acknowledges rewards for investments and the development of new business strategies. Dominant undertakings have the right to decide with whom they want to share “facilities”, Case C 7-97 *Oscar Bronner* [1998] ECR I-7791, ECLI:EU:C:1998:569, para. 26.

20 Flood (2016), 885. The effect of controlling hardware is discussed below.

21 Tampering with or removing DRM is treated as infringement under Directive 2001/29/EC of the European Parliament and of the Council of 22 May 2001 on the harmonisation of certain aspects of copyright and related rights in the information society OJ L 167, 22/06/2001 p. 0010–0019, Arts. 6, 7.

22 Flood (2016); Gaudin and White (2014); Gilbert (2015); Harrill (2013).

23 Some Member States have fixed book prices which breaks this dynamic, but the popularity of Amazon and its preference to have uniform contracts still affects these markets too.

24 Gilbert (2015), p. 177.

25 Some Member States prohibit a deviation from the RRP under rules focusing on cultural policy.

26 Gilbert (2015), p. 169.

27 Flood (2016), p. 885.

28 Harrill (2013), p. 200.

29 Kirkwood (2014), p. 39.

30 Kirkwood (2014); Harrill (2013).

31 Kirkwood (2014), p. 9.

32 Flood (2016), p. 886; Gilbert (2015), p. 169.

33 Harrill (2013), p. 201

34 Gilbert (2015), p. 166.

35 Rankin (2014); Ellis-Petersen (2014). Bloomsbury and the Independent Publisher Group had similar disputes.

36 Kirkwood (2014), p. 12.

37 Flood (2016).

38 Harrill (2013), p. 192; Kirkwood (2014), p. 18.

39 Price MFN and Retail Price MFN were banned to “eliminate the incentive for other publishers to have retailers on the agency model”. Summary of Commission decision of 12 December 2012 (*Case COMP/39.847 – E-Books*), OJ C 73/17, para. 28 https://eur-lex.europa.eu/legal-content/EN/TXT/PDF/?uri=CELEX:52013XC0313(03)&from=EN. Summary of Commission of 25 July 2013, Case *COMP/39.847/E-BOOKS*, OJ C 378/25 https://eur-lex.europa.eu/legal-content/EN/TXT/PDF/?uri=CELEX:52013XC1224(04)&from=EN.

40 Summary of Commission decision of 4 May 2017 relating to a proceeding under Art. 102 of the Treaty on the Functioning of the European Union and Art. 54 of the EEA Agreement (Case AT.40153 – *E-Book MFNS and related matters*) (notified under document C(2017) 2876) https://eur-lex.europa.eu/legal-content/EN/TXT/HTML/?uri=CELEX:52017XC0811(02)&from=EN; Full decision https://ec.europa.eu/competition/antitrust/cases/dec_docs/40153/40153_4392_3.pdf.

41 Kirkwood (2014), p. 50.

42 Harrill (2013), p. 193; Gilbert (2015), p. 181.

43 Directive 2001/29/EC of the European Parliament and of the Council of 22 May 2001 on the harmonisation of certain aspects of copyright and related rights in the information society OJ L 167, 22/06/2001 P. 0010–0019, chapter 3.

44 Directive 2001/29/EC of the European Parliament and of the Council of 22 May 2001 on the harmonisation of certain aspects of copyright and related rights in the information society OJ L 167, 22/06/2001 P. 0010–0019, chapter 4.

45 Directive 2001/29/EC of the European Parliament and of the Council of 22 May 2001 on the harmonisation of certain aspects of copyright and related rights in the information society OJ L 167, 22/06/2001 P. 0010–0019, Art. 6(4)

46 For a comprehensive evaluation, *see* Perzanowksi et al. (2016).

47 Elkin-Koren and Salzberger (2015), pp. 157–158.

48 The European Commission adopted decisions against major publishers under Art. 101 TFEU in 2012 and 2013 that retail price MFNs in their e-book publishing agreements must be removed, (*supra* note 38).

49 Gilbert (2015), p. 169.

50 Statista (2021).

51 Littlechild (2018), p. 211.

52 Rickets (2006), p. 45.

53 Harker and Kreutzmann-Gallasch (2016), p. 236.

54 Statista (2019b), pp. 85–86.

55 Some European countries (*e.g.* Austria, Bulgaria, parts of Belgium, France, Germany, Hungary, Italy, Luxembourg, the Netherlands, Portugal, Slovenia and Spain) have adopted a type of resale price maintenance or RPM regulation but most of these countries have chosen not to apply RPM regulation to e-books, Marcowitz-Bitton and Nussim (2020), pp. 852–854; *See also*, Monopolkommission (2018), pp. 29–30.

56 Littlechild (2018), p. 218.

57 Hovenkamp (2021), p. 1904.

58 It must be noted that some Member States have adopted national legislation regulating the accessibility of e-books. However, the scope of the national legislation is narrow and/or incomplete. For example, in Italy, the legislation is restricted to educational textbooks, European Commission, 2015. In France, legislation was adopted that addressed interoperability through DRM protections. It required that DRM protection should not restrict interoperability and providers are obliged to supply the necessary information, Code De La Propriété Intellectuelle, Art. L 331-5. The company can restrict the number of copy when making the information available, Art. 331-7. Although the French legislation addresses interoperability, it does not address the underlying problem of Amazon’s market power. However, it is argued that the French legislation overall is not effective as it provides too many exceptions, *see* Mazziotti (2008), pp. 195–196.

59 Consolidated Version of the TFEU, OJ C 326, 26 October 2012, pp. 47–390, Art. 102(a).

60 Case 27/76, *United Brands v. Commission*, [1978] ECR 207. ECLI:EU:C:1978:22, para. 65.

61 O’Donoghue and Padilla (2013), pp. 94–95.

62 Connolly 2021.

63 European Commission 1997, p. 5.

64 The European Commission concluded in its decision of 4 May 2017 Case AT.40153 – *E-book MFNs and related matters (Amazon)* that consumers do not regard e-books as substitutes for print books, (*supra* note 1) para. 43.

65 O’Donoghue and Padilla (2013), p. 139.

66 Case 27/76, *United Brands v. Commission*, [1978] ECR 207, ECLI:EU:C:1978:22, para. 39.

67 Amazon launched its digital stores in the United Kingdom and Germany in 2008, followed by Italy, Spain, The Netherlands, Sweden and Poland, Ecommerce News, “Amazon in Europe”, https://ecommercenews.eu/amazon-in-europe/.

68 This is in line with the European Commission’s findings in Case AT.40153. It should be noted, however, that the Commission refrains from taking a final decision on this matter, European Commission decision of 4 May 2017 Case AT.40153 – *E-book MFNs and related matters (Amazon)*, paras. 48–51. Fletcher and Lyons have shown that the Commission appears to adopt a wider approach in matters concerning technological products, Fletcher and Lyons (2016), pp. 21–22.

69 Case 27/76, *United Brands v. Commission*, [1978] ECR 207, ECLI:EU:C:1978:22, paras. 65–66.

70 Monti (2001).

71 *See e.g.*, Case 85/76 *Hoffmann-La Roche v. Commission*, [1979] ECR 461, ECLI:EU:C:1979:36, para. 39; And more recently, Case T-814/17, *Lietuvos geležinkeliai AB v. European Commission* (18 November 2020), ECLI:EU:T:2020:545, para. 346.

72 *See e.g.*, Case C-62/86 *Akzo v. Commission* [1991], ECR I-3359, ECLI: EU:C:1991:286, para. 60.

73 Joined Cases T-191-98, T212/98 to T/214/08 *Atlantic Container Line and Others v. Commission*, [2003], ECR II-3275, ECLI:EU:T:2003:245, para. 209; Case T-336/07 *Telefónica SA v. Commission*, ECLI:EU:T:2012:172, para. 150.

74 The European Commission outlined in its 2009 Communication that it is unlikely that a company holding a market share of 40% or less is dominant. European Commission, 2009, para. 14.

75 *See*, Case 85/76 *Hoffmann-La Roche v. Commission*, [1979] ECR 461, ECLI:EU:C:1979:36, paras. 42 and 47; Case T-219/99 *British Airways plc v. Commission,* [2003], ECR II-5917, ECLI:EU:T:2003:343, para. 210.

76 Statista (2019b), pp. 85–86. It must be noted that the market share is based on online purchases by provider. Consumers may have purchased e-books from more than one provider.

77 Statista (2019b), pp. 85–86.

78 Case 85/76 *Hoffmann-La Roche v. Commission* [1979] ECR 461, ECLI:EU:C:1979:36, para. 48.

79 European Commission, decision 2017 (*supra* note 1), para. 17.

80 *See*, Case T-201/04 *Microsoft v. Commission* [2007] ECR II-3601, ECLI:EU:T:2007:289. Commission decision of 04.07.2004 (Case COMP/38.784 *– Wanadoo España v. Telefónica*), para. 226.

81 *See*, Case T-201/04 *Microsoft v. Commission* [2007] ECR II-3601, ECLI:EU:T:2007:289. Commission decision of 04.07.2004 (Case COMP/38.784 – *Wanadoo España v. Telefónica*), para. 225.

82 This is in line with the European Commission’s view, European Commission, decision 2017 (*supra* note 1), para. 65.

83 Bläsi and Rothlauf (2013), pp. 18–23.

84 Gilbert (2015), p. 172.

85 *See*, *e.g.*, Waddams Price and Zhu (2016), pp. 134–138.

86 European Commission 1997, p. 5.

87 European Commission, decision 2017 (*supra* note 1), para. 65(2).

88 For an overview of potential barriers to entry and features of the market and the concerned undertaking as well as the relevant case law, *see* O’Donoghue and Padilla, pp. 152–166. The Commission’s inquiry into the German and English e-books sector has revealed that Amazon gained market power in that segment to the detriment of its competitors, European Commission, decision 2017 (*supra* note 1), para. 65(3).

89 Case 85/76 *Hoffmann-La Roche v. Commission*, [1979] ECR 461, ECLI:EU:C:1979:36, para. 91; Case C-62/86 *Akzo v. Commission* [1991], ECR I-3359, ECLI: EU:C:1991:286, paras. 69–70.

90 For an overview of the various parity clauses used by Amazon, *see* European Commission, decision 2017 (*supra* note 1), pp. 9–13.

91 European Commission, decision 2017 (*supra* note 1), paras. 3, 8–9. This is beyond the scope of this article, but it should be noted that in the US, the combability of MFNs and price parity clauses were also part of a competition investigation against Amazon by the Committee of the Judiciary, Committee of the Judiciary (2020), pp. 295–296.

92 European Commission, decision 2017 (*supra* note 1), paras. 74–89 and 91–105.

93 European Commission, decision 2017 (*supra* note 1), paras. 115–144.

94 European Commission, decision 2017 (*supra* note 1), para. 129.

95 European Commission, decision 2017 (*supra* note 1), para. 151.

96 Case COMP/39.847 – *E-Books*, OJ C 73/17. More recently national competition authorities investigated the use of MFN in agreements between price comparison operators and suppliers as well as retailers and online marketplaces. A discussion of these cases is beyond the scope of this article. For an assessment and summary of these cases, *see* Akman (2016); Chappatte and O’Connel (2020); Bostoen (2017). These cases show that price MFN can be distinguished between “wide” and “narrow” MFNs. “Wide” MFNs require that the price offered on the comparison platform cannot be higher than on any other website; “narrow” MFNs require that the price on the comparison website must be at least the same price as the price offered on the supplier’s website, it can also be lower. For the definitions, *see* Bostoen, pp. 225–226. The Commission did not distinguish between “wide” and “narrow” MFNs in the *Amazon* e-book decision. Still, the MFNs enforced by Amazon in its e-book agreements would fall in the “wide” MFN category as Amazon required publishers or intermediaries to offer at least the same conditions that they would offer competing firms, Bostoen, pp. 233–234. For an overview of all the MFNs used in the e-book agreements, *see* European Commission, decision 2017 (*supra* note 1), pp. 22–38.

97 Bostoen (2017), p. 223. Bostoen defines online platform as “intermediaries operating in multi-sided markets, in which they seek to facilitate interactions between different user groups (the “sides” of the market), p. 224.

98 European Commission, Commission decision of 27.6.2017 relating to proceedings under Art. 102 of the Treaty on the Functioning of the European Union and Art. 54 of the Agreement on the European Economic Area (Case AT.39740 – *Google Search (Shopping)*, https://ec.europa.eu/competition/antitrust/cases/dec_docs/39740/39740_14996_3.pdf. On appeal, the General upheld the Commission decision, Case T-612/17 *Google Alphabet v. Commission (Google Shopping)* [2021], ECLI:EU:T:2021:763.

99 Akman (2016), p. 823.

100 European Commission (2017), p. 180.

101 Apple imposes the obligation on app developers to provide their apps through the Apple AppStore. Apple charges a commission fee which is in most cases passed on to the consumer. Please note that a discussion of the *Apple AppStore* case is beyond the scope of this paper. European Commission, Case AT.40437 *Apple – AppStore Practices (music streaming)* https://ec.europa.eu/competition/antitrust/cases/dec_docs/40437/40437_657_5.pdf and Case AT.40652 – *Apple – AppStore Practices (e-books/audiobooks)* https://ec.europa.eu/competition/antitrust/cases/dec_docs/40652/40652_142_3.pdf; Press Release (16 June 2020) “Antitrust: Commission opens investigations into Apple’s App Store rules”, https://ec.europa.eu/commission/presscorner/detail/en/ip_20_1073.

102 The commitments expire in May 2022. European Commission, decision 2017 (*supra* note 1), Art. 1.

103 European Commission, decision 2017 (*supra* note 1), pp. 26–31.

104 For a discussion of Amazon’s market share in the Europe sector, *see* above 3.2.

105 *See e.g.*, Case C-209/10 *Post Danmark A/S v. Konkurrencerådet*, [2012], ECLI:EU:C:2012:172, para. 23; Case C-202/07 P *France Télécom SA v. Commission*, [2009] ECR I-2369, ECLI:EU:C:2009:214, para. 105.

106 Nihoul (2012), pp. 55, 64.

107 European Commission (2009), para. 19. Please note that the authors are aware that the term “consumer” covers all parties that are affected by the anticompetitive behaviour of the undertaking, not just the end-consumer, European Commission 2004, para. 84.

108 European Commission, decision 2017 (*supra* note 1), para. 172. However, the Commission concluded that this went “beyond the competition concerns by the Commission in its Preliminary Assessment and therefore does not need to be addressed in the Final Commitments,” Commission decision of 4 May 2017 relating to a proceeding under Art. 102 of the Treaty on the Functioning of the European Union and Art. 54 of the EEA Agreement (Case AT.40153 – *E-Book MFNS and related matters*) (notified under document C(2017) 2876), para. 197.

109 Case C 7-97 *Oscar Bronner* [1998] ECR I-7791, ECLI:EU:C:1998:569, para. 26; *See also* Ullrich (2012), pp. 4, 22.

110 Case T-201/04 *Microsoft v. Commission* [2007] ECR II-03601, ECLI:EU:T:2007:289, para. 331; Joined Cases C-241/91 P and C-242 *Radio Telefis Eireann (RTE) and Independent Television Publications (ITP) v. Commission of the European Communities* (*Magill*), [1995] ECR I-743, ECLI:EU:C:1995:98, para. 50.

111 Commission decision of 24 May 2004 relating to a proceeding pursuant to Art. 82 of the EC Treaty and Art. 54 of the EEA Agreement against Microsoft Corporation (Case Comp/C-3/37.792 – *Microsoft*) (notified under document number C(2004) 900), OJ L 32/23 (*Microsoft* decision). In *Microsoft*, two separate abuses were addressed: first, interoperability – the refusal to supply proprietary information; the second abuse concerned the tying of the Microsoft Windows Media Player. This article only focuses on the first abuse – refusal to supply information.

112 “Work group server operating systems” are systems that can provide basic services used by offices so that they could, for example, share files within their network, *Microsoft* decision (*supra* note 105), para. 7.

113 *Microsoft* decision (*supra* note 112), para. 18.

114 *Microsoft* decision (*supra* note 112), para. 2.

115 Case T-201/04 *Microsoft v. Commission* [2007] ECR II-03601, ECLI:EU:T:2007:289.

116 Joined Cases C-241/91 P and C-242 *Radio Telefis Eireann (RTE) and Independent Television Publications (ITP) v. Commission of the European Communities* (*Magill*), [1995] ECR I-743, ECLI:EU:C:1995:98. In *Magill*, a new entrant (Magill) wanted to publish a weekly television guide; the information was copyright protected. The incumbent national broadcasters refused to grant an intellectual property licence to Magill, paras. 7–10. The Commission found that the national broadcasters had abused their dominant position. The Court held that the refusal to grant a licence does not in itself is enough for an abuse, para. 49. However, the broadcasters were the only right holders and by not providing the information, Magill was excluded from the aftermarket and there was no other objective justification, paras. 53–56. Thus, there were “exceptional circumstances.” *IMS Health* was a later case where the Court of Justice again had to examine the validity of refusal to licence, Case C-418/01 *IMS Health Gmbh & Co. OHG v. NDC Health NDC Health GmbH & Co. KG*, [2004] ECR-I 5039, ECLI:EU:C:2004:257. The Court of Justice elaborated on *Magill* and confirmed that in cases where the intellectual property is indispensable for offering a new product the refusal to supply the property “constitutes an abuse of a dominant position within the meaning of Art. 82 EC [now Art. 102 TFEU] where the following conditions are fulfilled: – the undertaking which requested the licence intends to offer, on the market for the supply of the data in question, new products or services not offered by the owner of the intellectual property right and for which there is a potential consumer demand; – the refusal is not justified by objective considerations; – the refusal is such as to reserve to the owner of the intellectual property right the market for the supply of data on sales of pharmaceutical products in the Member State concerned by eliminating all competition on the market”, [para. 52].

117 Case T-201/04 *Microsoft v. Commission* [2007] ECR II-03601, ECLI:EU:T:2007:289, paras. 332–333.

118 The other requirements for an infringement of Art. 102 TFEU, such as the relevant product and geographic market and dominance have been examined, *see* above “3.2 Relevant Market, Dominance and Abuse”.

119 In *Microsoft*, the Commission also aspired a higher degree of interoperability (client/server interoperability and server/server interoperability) and the CFI agreed with the Commission’s line of reasoning, Case T-201/04 *Microsoft v. Commission* [2007] ECR II-03601, ECLI:EU:T:2007:289, paras. 374, 382, 386.

120 The General Court held in *Google Shopping* that indispensability is not required. Yet, this case must be distinguished from the e-book scenario due to the functioning of the search engine. Search engines are capable of including results from their firm as well as third-party results at the same time (“Google’s general results page has characteristics akin to those of an essential facility”) but Google always favoured its own results which constitutes an abuse, Case T-612/07 *Google and Alphabet v. Commission* [2021] ECLI:EU:T:2021:763 (not yet reported), paras. 178, 212–248. The difference between Google Shopping and Amazon is that in Google Shopping, a “reactive intervention” (the undertaking concerned must only be ordered to stop) was sufficient to end the abuse; the case of Amazon’s Kindle requires a “proactive intervention” to end the abuse – the competition authority must prescribe an additional measure or conditions, Colomo (2019), pp. 548, 550.

121 The CFI found that Windows was with a market share of 90% the “de facto” PC operating system, Case T-201/04 *Microsoft v. Commission* [2007] ECR II-03601, ECLI:EU:T:2007:289, paras. 387–392.

122 Case T-201/04 *Microsoft v. Commission* [2007] ECR II-03601, ECLI:EU:T:2007:289, paras. 523–525, 669

123 It was shown above that in 2019, Amazon has an estimated market share between 62–73% in Germany, France, Italy and Spain and 84% in the United Kingdom, *see* above “3.2. Relevant Market, Dominance and Abuse”.

124 European Commission, decision 2017 (*supra* note 1), para. 65(2).

125 *See* for concept, Case 85/76 *Hoffmann-La Roche v. Commission*, [1979] ECR 461, ECLI:EU:C:1979:36, para. 41.

126 Bittar (2015), pp. 24–26.

127 Nazzini 2008, p. 60.

128 Mazzoli EM 2021. Mazzoli points out that the lack of available data makes it difficult to develop an approach that benefits smaller independent players in the market. The Covid-19 pandemic has increased Amazon’s sales and strengthened its position in the market.

129 Case T-201/04 *Microsoft v. Commission* [2007] ECR II-03601, ECLI:EU:T:2007:289, para. 643. Drexl (2017) suggests that the “new product rule” in *Microsoft* is satisfied when the lack of supply negatively affects innovation, p. 284.

130 European Commission, decision 2017 (*supra* note 1), para. 101. *See also*, Case T-201/04 *Microsoft v. Commission* [2007] ECR II-03601, ECLI:EU:T:2007:289, para. 694.

131 European Commission, decision 2017 (*supra* note 1), para. 102. The Commission’s findings relate to the use of MFN clauses; the difficulties and the costs also arise due to the lack of standardization.

132 Case T-201/04 *Microsoft v. Commission* [2007] ECR II-03601, ECLI:EU:T:2007:289, paras. 709–729; Nazzini (2008), p. 61. LaRouche argues that the long-term effects must be considered, p. 947.

133 LaRouche (2009), p. 947

134 Art. 7(1) of Regulation 1/2003. An affirmative measure was also imposed on the dominant undertakings in *Microsoft – abuse concerning the issue of interoperability, Magill* and *IMS Health*.

135 *See*, *e.g.*, Case C-426/93 *Germany v. Council* [1995] ECR I-3723, ECLI:EU:C:1995:367, para. 42.

136 O’Donoghue and Padilla, p. 883.

137 Forrester (2004) argues that the Commission also uses Art. 102 TFEU (then Art. 82) as a regulatory tool to achieve policy objectives, pp. 922 and 951. *See also* O’Donoghue and Padilla, p. 883.

138 Standardization, on the one hand, can increase innovation and the compatibility between the different products gives consumers more choice and enhance consumer welfare. On the other hand, standardization can create a barrier to entry where a firm is excluded from getting access to the standard or promote collusion and higher costs for consumers, European Commission (2011) paras. 263–269.

139 The degree to which piracy replaces sales or facilitates them is highly debated and beyond the scope of this article.

140 A simple Google search for nearly any book title with PDF or EPUB added will give results where the book can be downloaded for free. This includes either by hosting, online storage lockers or torrents.

141 35% of surveyed UK users have illegally accessed a book in March 2019, (Statista (2019a), p. 12).

142 Statista (2019a), p.22: in the UK, 70% of surveyed individuals have bought printed books through Amazon, significantly higher than No. 2 Waterstones (39%) and WH Smith (30%) in 2019–2020.

143 There are no systematic complaints about Amazon in the context of book piracy that the author has been able to locate. Some anecdotal evidence, *see* for example Bailey (2016), exists but it is not anywhere on the same scale as the issues on YouTube for example and has not entered the public debate on a wide scale.

144 Amazon has bought some back catalogues, including works by Iain Flemming, but this is the exception. The rights are usually held by the publishers or the authors. Harrill (2013), p. 201.

145 The A9 algorithm is developed by a subsidiary of Amazon, emphasising sales conversion. This means that books that sell well are ranked higher and therefore get more traffic, re-enforcing the sales pattern. As a side effect, not all listings need to be policed but the higher-ranking ones as overly successful pirate editions float to the top by themselves and otherwise remain buried down the list, de facto unidentifiable for most users.

146 Lamping (2015), p. 141.

## References

[CR1] Adeney E (2006). The moral rights of authors and performers: an international comparative analysis.

[CR2] Akman P (2016). A competition law assessment of platform most-favoured-customer clauses. J Compet Law Econ.

[CR3] Amazon (2021) E-book bestseller list (paid). https://www.amazon.co.uk/Best-Sellers-Kindle-Store/zgbs/digital-text/ref=zg_bs_nav_0

[CR4] Australian Government – Productivity Commission (2016) Intellectual Property Arrangement – Inquiry Report No 78. https://www.pc.gov.au/inquiries/completed/intellectual-property/report

[CR5] Bailey J (2016) Amazon has a serious copyright problem – cutting down the amazon forest. Plagiarism Today. https://www.plagiarismtoday.com/2016/06/16/amazon-serious-copyright-problem/

[CR6] Barnett M (2014). Copyright without creators. Rev Law Econ.

[CR7] Bittar AC (2015) Unlocking the bates of Alexandria: DRM, competition and access to e-books. https://www.plagiarismtoday.com/2016/06/16/amazon-serious-copyright-problem/

[CR8] Bläsi C and Rothlauf F (2013) On the interoperability of e-book formats. European and International Booksellers Federation, Brussels. https://www.booksellers.org.uk/BookSellers/BizFormFiles/936121cb-a426-46da-b9aa-db8bd285d21e.pdf

[CR9] Bostoen F (2017). Most favoured nation clauses: towards an assessment framework under EU competition law. Eur Compet Regul Law Rev.

[CR10] Caves R (2000). Creative industries – contracts between art and commerce.

[CR11] Chappatte P, O’Connell K (2020) European Union – e-commerce: most favoured nation clauses. Global Competition Review. https://globalcompetitionreview.com/guide/e-commerce-competition-enforcement-guide/third-edition/article/european-union-e-commerce-most-favoured-nation-clauses

[CR12] Colomo PI (2019). Indispensability and Abuse of Dominance: From Commercial Solvents to Slovak Telekom and Google Shopping. J Eur Compet Law Pract.

[CR13] Columbia Law School, Keep your copyright – “out of print” clauses. https://web.law.columbia.edu/keep-your-copyrights/copyrights/out-of-print-clauses

[CR14] Committee of the Judiciary (2020) Investigation of competition in digital market. Majority Staff Report, Subcommittee on Antitrust, Commcercial and Administrative Law of the Committee on the Judiciary. https://judiciary.house.gov/uploadedfiles/competition_in_digital_markets.pdf?utm_campaign=4493-519

[CR15] Conolly B (2021) Top Amazon product categories 2021. https://www.junglescout.com/blog/amazon-product-categories/

[CR16] Davies G, Garnett K (2016). Moral rights.

[CR17] Drexl J (2017). Competitive markets for industrial data – between propertisation and access. J Intell Prop Tech & Elec Com L.

[CR18] Ecommerce News. Amazon in Europe. https://ecommercenews.eu/amazon-in-europe/

[CR19] Elkin-Kiren N, Salzberger E (2015). The law and economics of intellectual property in the digital age.

[CR20] Ellis-Petersen H (2014). Amazon and publisher Hachette end dispute over online book sales. The Guardian. https://www.theguardian.com/books/2014/nov/13/amazon-hachette-end-dispute-ebooks

[CR21] European Commission (2004) Guidelines on the application of Article 81(3) of the Treaty, (Communication), Guidelines on the application of Article 81(3) of the Treaty. C 101/97. https://eur-lex.europa.eu/LexUriServ/LexUriServ.do?uri=OJ:C:2004:101:0097:0118:EN:PDF

[CR22] European Commission (2009) Guidance on the Commission’s enforcement priorities in applying Article 82 of the EC Treaty to abusive exclusionary conduct by dominant undertakings. OJ C 45/7 https://eur-lex.europa.eu/legal-content/EN/TXT/PDF/?uri=CELEX:52009XC0224(01)&from=EN

[CR23] European Commission (1997) Notice on the definition of relevant market for the purposes of Community competition law. Document 31997Y1209(01). OJ C 372

[CR24] European Commission (2011) Guidelines on the applicability of Article 101 of the Treaty on the Functioning of the European Union to horizontal co-operation agreements (Communication) OJ C 11, pp 1–72. https://eur-lex.europa.eu/legal-content/EN/ALL/?uri=CELEX%3A52011XC0114%2804%29

[CR25] European Commission (2017) Final report on the E-commerce Sector Inquiry (Commission Staff Working Document) SWD(2017) 154 final. https://ec.europa.eu/competition/antitrust/sector_inquiry_swd_en.pdf

[CR26] European Commission (2021) Antitrust: Commission sends statement objections to apple on app store rules for music streaming providers. Press Release, https://ec.europa.eu/commission/presscorner/detail/en/IP_21_2061

[CR27] Fletcher A, Lyons B (2016) Geographic market definition in European Commission merger control. A study for DG competition. Centre for Competition Policy, University of East Anglia, Norwich. https://ec.europa.eu/competition/publications/reports/study_gmd.pdf

[CR28] Flood Z (2016). Antitrust enforcement in the developing e-book market: Apple, Amazon, and the future of the publishing industry. Berkeley Technol Law J.

[CR29] Forrester IS (2004). Article 82: remedies in search of theories?. Fordham Int Law J.

[CR30] Garon JM (2013). Digital Hollywood 2.0: reimagining film, music, television, and publishing distribution as a global artist collaborative. Mich St Int'l L Rev.

[CR31] Gaudin G, White A (2014). On the antitrust economics of the electronic books industry. DICE Discussion Paper.

[CR32] Geiger C, Gervais D, Senftleben M (2013). The three-step test revisited: how to use the test’s flexibility in national copyright law. Am Univ Int Law Rev.

[CR33] Gilbert R (2015). E-books: a tale of digital disruption. J Econ Perspect.

[CR34] Harker M, Kreutzmann-Gallasch A (2016). Universal service obligations and the liberalization of network industries: taming the Chimera?. Eur Compet J.

[CR35] Harrill J (2013). Two years is too long: the two-year band on the agency model can save the e-book industry but ruin bookstores. Northern IL U Law Rev.

[CR36] Hovenkamp H (2021) Antitrust and platform monopoly. Yale L.J. 130. https://scholarship.law.upenn.edu/faculty_scholarship/2192

[CR37] Johnson JP (2017). The agency model and MFN clauses. Rev Econ Stud.

[CR38] Kirkwood J (2014). Collusion to control a powerful customer. Univ Miami Law Rev.

[CR1000] Lamping M, Hilty R, Liu KC (2015). Refusal to licence as an abuse of market dominance: from commercial solvents to microsoft. Compulsory licensing. MPI studies on intellectual property and competition law.

[CR39] LaRouche P (2009). European Microsoft case at the crossroads of competition policy and innovation: comment on Ahlborn and Evancs. Antitrust Law J.

[CR40] Littlechild S (2018). Regulation and the nature of competition. J Air Transp Manag.

[CR41] Mandal S (2021) An introduction to the most common eBook formats currently in use (Blog “Good E Reader”). https://goodereader.com/blog/e-book-news/an-introduction-to-the-most-common-ebook-formats-currently-in-use

[CR42] Marcowitz-Bitton M, Nussim J (2020). Regulation of book markets. Wash U L Rev.

[CR43] Mazziotti G (2008) Freedom of use vs DRM technology. In: EU digital copyright law and the enduser. Springer, Berlin, Heidelberg, pp. 179–229. 10.1007/978-3-540-75985-0_7

[CR44] Mazzoli, EM (2021) Book and publishing statistics in Europe: the need to improve a fragmented market with a lack of access to data. http://www.aldusnet.eu/k-hub/book-publishing-statistics-europe-need-improve-fragmented-market-lack-access-data/

[CR45] Monopolkommission (2018) Fixed book prices in a changing market environment. Special Report No 80 – Special Report by the Monopolies Commission pursuant to Section 44(1)(4) of the Act Against Restraints on Competition. https://www.monopolkommission.de/images/PDF/SG/s80_fulltext.pdf

[CR46] Monti M (2001) Market definition as a cornerstone of EU competition policy. Speech/01/439, Helsinki. https://ec.europa.eu/commission/presscorner/detail/en/SPEECH_01_439

[CR47] Nazzini R (2008). The Microsoft case and the future of Article 82. Antitrust.

[CR48] Nihoul P (2012). Freedom of choice: the emergence of a powerful concept in European competition law. Concurrences.

[CR49] O’Donoghue R, Padilla J (2013). The law and economics of Article 102 TFEU.

[CR50] Parc J, Messerlin P (2021). The true impact of shorter and longer copyright durations: from authors’ earnings to cultural creativity and diversity. Int J Cult Policy.

[CR51] Perzanowksi A, Schultz J, Denardis L, Zimmer M (2016). The end of ownership: personal property in the digital economy.

[CR52] Ramalho A (2014). Conceptualising the European Union’s competence in copyright – what can the EU do?. IIC.

[CR53] Rankin J (2014) Amazon and Hachette feud could rewrite the book on publishing. https://www.theguardian.com/technology/2014/aug/25/amazon-hachette-publishing-future-ebooks

[CR54] Reed A (2021) Apple is going to court with “Fortnite” and it could forever change how apps work. The Washington Post. https://www.washingtonpost.com/technology/2021/04/30/epic-apple-trial-antitrust-fortnite-app-store/

[CR55] Rickets M (2006) Economic regulation: principles, history, and methods. In: Crew M, Parker D (eds) International handbook on economic regulation. Edward Elgar, pp 34–62. 10.4337/9781847201614

[CR56] Ricketson S, Ginsburg JC (2006). International copyright and neighboring rights: the Berne Convention and beyond.

[CR57] Searle N, Brassel M (2016). Economic approaches to intellectual property.

[CR58] Statista (2019a) E-books in the United Kingdom (UK). https://www-statista-com.plymouth.idm.oclc.org/study/24380/e-books-in-the-united-kingdom-statista-dossier/

[CR59] Statista (2019b) Digital media report 2020. https://www.statista.com/study/44526/digital-media-report/

[CR60] Statista (2021) E-book worldwide report 2021. https://www.statista.com/outlook/dmo/digital-media/epublishing/e-books/worldwide

[CR61] Thompson J (2012). Merchants of culture – the publishing industry in the 21st century.

[CR62] Townley B, Roscoe P, Searle N (2019). Creating economy – enterprise, intellectual property and the valuation of goods.

[CR63] Ullrich H (2012) Intellectual property: exclusive rights for a purpose – the case of technology protection by patents and copyright. Max Planck Institute for Intellectual Property & Competition Law Research Paper No. 13-01. https://papers.ssrn.com/sol3/papers.cfm?abstract_id=2179511

[CR64] Waddams Price C, Zhu M (2016). Empirical evidence of consumer response in regulated markets. J Compet Law Econ.

